# The value of doing philosophy in mental health contexts

**DOI:** 10.1007/s11019-020-09961-4

**Published:** 2020-07-23

**Authors:** Sophie Stammers, Rosalind Pulvermacher

**Affiliations:** 1 The University of Birmingham, Birmingham, UK; 2 Independent Lived Experience Researcher, London, UK

**Keywords:** Mental health, Philosophy groups, Epistemic injustice, Stigma, Mental health advocacy

## Abstract

People experiencing mental distress and illness are frequently on the receiving end of stigma, epistemic injustice, and social isolation. A range of strategies are required to alleviate the subsequent marginalisation. We ran a series ‘philosophy of mind’ workshops, in partnership with a third-sector mental health organisation with the aim of using philosophical techniques to challenge mental health stigma and build resources for self-understanding and advocacy. Participants were those with lived experience of mental distress, or unusual beliefs and experiences; mental health advocates; and mental health service providers (such as counsellors, psychologists and psychiatrists). We draw on a shared perspective as a participant and facilitator of the workshop series to assess their impact. We discuss the following benefits: (i) the opportunity for structured discussion of experiences and models; (ii) dialogue across different mental health backgrounds; (iii) the potential to reduce self-stigma and to increase self-understanding and advocacy; and (iv) the potential to alleviate (some) epistemic injustice. We invite researchers and mental health practitioners to consider further opportunities to investigate the potential benefits of philosophy groups in mental health settings to establish whether they generalise.

## Background

In this paper, we report on a series of ‘philosophy of mind’ workshops, which were run in partnership with a third-sector mental health organisation during the winter of 2017–2018. We are the workshop series designer and facilitator (an academic philosopher working on an interdisciplinary research project on mental health at the time of the workshop series), and a workshop participant and presenter. Our perspective is informed by the lived experience of mental distress, and knowledge of philosophical approaches to mental health.^1^ This paper constitutes our shared reflections on the workshop series, and where we speak from our distinct perspectives and experiences, we sign-post this accordingly (e.g. “from the participant/facilitator perspective…”).

A number of background conditions motivate the project, including mental health stigma, epistemic injustice and social isolation. We briefly describe each in turn:

### Mental health stigma

People experiencing mental distress and illness are frequently on the receiving end of stigma, in which they are unjustifiably assumed to have, or otherwise associated with, negative characteristics. For instance, people are shown to associate mental illness with incompetence and being dangerous (Corrigan and Watson 2002; Phelan et al. 2000). The stigma around mental health is pervasive (e.g. Brener et al. 2013; Peris et al. 2008; and see Puddifoot 2019 for an overview). People who experience mental distress have been shown to stigmatise themselves (Corrigan et al. 2014; Sickel et al*.* 2019; Knaak et al. 2017; Bracke et al. 2019). These stereotypical associations tend to be reinforced by the media. For example, primetime television presents overwhelmingly negative portrayals of mental illness, with the characters who experience it being the most likely to commit violence (Signorielli 1989). Negative portrayals of mental illness across the media persist, and have been shown to contribute the public’s negative attitudes towards those who experience mental distress (Sieff 2003; Ma 2017). The stigma encountered in everyday life has been described as worse than the mental illness and distress itself (Thornicroft 2017).

### Epistemic injustice

Related to stigma, is the notion of epistemic injustice, a distinctive kind of injustice which harms someone by limiting their capacity to share or attain knowledge, on the basis of their perceived membership of a marginalised social group. Fricker articulates two chief kinds of epistemic injustice (Fricker 2007). There is testimonial injustice, in which the testimony of people from marginalised groups is not appropriately believed. There is also hermeneutic injustice, in which marginalised groups have fewer resources for understanding aspects of their social experiences in comparison to groups with greater social and cultural power. Multiple authors have investigated how people in mental distress are at risk of epistemic injustice in both medical and social encounters (Carel and Kidd 2014; Sanati and Kyratsous 2015; Wardrope 2015; Crichton et al. 2017; Kurs and Grinshpoon 2018; Miller Tate 2018). Richard Lakeman (2010), a mental health professional, gives an account of personal experience of epistemic injustice, in which he noticed a marked difference in how his testimony was received by colleagues during a period of mental distress as compared to before that period. In particular, he reports that his testimony regarding reactions he was having to a prescribed psychotropic drug was not taken as seriously by his colleagues as they had taken his testimony prior to this period of mental distress.

### Social isolation

Zavaleta et al. describe social isolation as an “inadequate quality and quantity of social relations with other people at the individual, group, community, and larger social environment levels where human interaction takes place,” (Zavaleta et al. 2014; in Wang et al. 2017). Those experiencing mental distress and illness also frequently experience social isolation (White et al. 2000; Cacioppo et al. 2006; Richman and Sokolove 1992). From the perspective of lived experience, we suggest that social isolation is importantly connected to the above two features. Experiencing systematic social injustice in daily life compounds the sense of social isolation felt in mental distress, and can become part of mental distress itself. Meanwhile, taking action to tackle stigma takes up a lot of emotional energy, particularly when it is done as an individual as opposed to when part of a collective, and can result in exhaustion, which itself compounds social isolation.

Numerous authors have considered a variety of means of alleviating the negative consequences of stigma, epistemic injustice, and social isolation in mental health contexts (e.g. Thornicroft et al. 2016; Crichton et al. 2017; Evans-Lacko et al. 2014). It is likely that a combination of strategies are required to make a positive impact. In this paper, we explore whether doing philosophy together might present one such means of making a modest but positive impact.

## Philosophy groups and mental health

The project took place between October 2017 and January 2018, in partnership with Mind in Camden, a mental health charity based in London, UK. Participants were enrolled in the free, six-session workshop series, after responding to an email announcement on the Mind in Camden mailing list, resulting in two groups each comprising ten participants. Participants drew on a variety of backgrounds in mental health. The groups included people with lived experience of mental distress, or cognitions which attract a psychiatric diagnosis; mental health advocates; and mental health service providers (such as counsellors, psychologists and psychiatrists). Many people belonged to more than one of these categories.

The recognition that pronouncements in mental health contexts should be open to philosophical scrutiny was a key motivator for our philosophy groups. A chief aim was to critically explore the association between irrationality and mental illness. As Elly Vintiadis observes, attention to the manuals which set the diagnostic criteria for mental disorders reveals that “rationality plays a big role in what counts as a mental disorder and, hence, in who we judge to have one and how we treat them” (Vintiadis 2016). We explored empirical evidence of irrational cognitions in the non-clinical population which are not routinely pathologised. We then used philosophical enquiry to investigate the similarities and differences between these and cognitions in mental distress (which are routinely pathologised), in order to critically investigate the role of rationality in the ascription of pathology.

Sessions were 90 min long.^2^ The six sessions were structured around the following topics: (1) Introduction and philosophical techniques; (2) Experiences; (3) Beliefs; (4) Rationality; (5) Models of mental health; (6) Evaluation (recapping learning from previous five sessions, critical analysis of value judgements made about different experiences and beliefs). In developing these workshops, we also created open-access resources for facilitating them, which can be accessed online.^3^ There, a detailed description of all sessions and content is available, as well as a facilitator pack, but to give some idea, we describe a few of the sessions in brief here.

The first session introduced the notion of philosophical argument: for instance, articulating a claim and supporting it with evidence, distinguishing normative from descriptive claims; and recognising and scrutinising underlying assumptions. Participants also split into groups to discuss different examples of thought experiments provided by the facilitator (e.g. the glass bridge from Gendler (2008) and the city dweller from Tufayl (1972), and learned about how to use them to tease out intuitions and to support arguments by coming up with their own thought experiments. We focused on philosophy as a dialectical practice that involves at least two parties exchanging ideas. Each party can aim to convince the other of their view, or, as we preferred to present the practice: each person uses this opportunity to articulate their view by trying make their point as clearly as possible, and in doing so, perhaps understands their own thought process on the matter at hand a little better. Participants’ positions evolve as the clarity of their thinking increases, with some who start out on one side of the debate ending up on the other at the end of the practice.

The second session opened with a general discussion, in which participants considered the nature of experience, and what (if anything) is special about experiences termed “hallucinations”. Guided by the facilitator, participants discussed two models: passive processing, in which the content of experience is accounted for by input from the external world, and predictive processing, in which a variety of internal processes also shape experiential content. The facilitator then presented a series of optical illusions shown via a slide deck (but which could also be shown with hand-outs), in order to demonstrate how internal processes sometimes shape experiential content, even for experiences not typically considered to be pathological. For instance, to provoke thought and discussion on the latter, the facilitator shared a series of images which appeared initially to be nebulous and unorganised shapes, but which could be perceived as an object, only after one is presented with an idea of what to look for. Guided by the facilitator, participants discussed Andy Clark’s notion of perception as “controlled hallucination” (2013). Participants then discussed whether systematic distinctions can be made between, for instance, hallucinations and voice hearing that sometimes accompany a diagnosis of schizophrenia, and experiences of optical and auditory illusions in people without this diagnosis, and what these distinctions might consist in.

The following sessions proceeded with a similar structure: (i) an open discussion regarding the phenomena to be investigated that week (e.g. belief and delusion; rationality); (ii) an overview of competing philosophical models of the phenomena, guided by the facilitator; (iii) discussion of examples (provided by the facilitator); and (iv) critical analysis of the models in light of the examples discussed. The sessions involved conceptual analysis, with participants considering, for instance, whether the concept of a hallucination is accurately captured in the definitions given by various researchers (for example, Cachia et al*.* say “visual hallucinations [are] defined as erroneous visual perceptions not elicited by an external stimulus” (2015: 1101)). Participants also considered whether psychiatric concepts, such as that of a delusion, are accurately described in various iterations of diagnostic manuals [e.g. the *Diagnostic and statistical manual of mental disorders* (American Psychiatric Association (2013)]. The sessions also involved discussion of empirical findings. For instance, in the third session, we compared findings regarding what have been termed “grandiose delusions” about having inflated self-worth (Knowles et al. 2011); with empirical findings regarding inflated self-worth in the non-clinical population, such as Cross’s finding that 94% of the college professors believe that they do above-average work (1977). Some sessions also involved discussions of individual cases, such as of individuals with schizophrenia and dementia, for the purpose of contextualising the associated unusual or ungrounded cognitions in the context of the person’s life experiences and relationships.^4^ This facilitated discussion of how these cognitions can be both harmful and helpful (in so far as they can help to make sense of that person’s world in times of difficulty, and can enhance agency, motivation and sociability).

As is perhaps evident from the above, the content of the workshop series goes beyond what analytic philosophers might recognise as part of a typical philosophy of mind course, involving discussion of topics in epistemology (such as the nature of belief and rationality); the philosophy of cognitive science (such as the comparative analysis of empirical findings in the mind sciences and clinical psychology); and social and political philosophy, particularly in session five, which was focused on different models of mental health, with discussion of cultural concepts of illness and wellness, wider societal circumstances that contribute to mental distress, and power dynamics inherent in clinical interactions. Since all discussions involved some focus on the mind and different perceptions of thinking, we settled for the name “philosophy of mind” for the workshop series, to reduce jargon and so as not to alienate those who had not studied philosophy before.

Participation was not premised on any philosophical training, and whilst some participants were attracted to the workshops because they had previously studied philosophy in some format, for others, the groups constituted their first engagement with philosophy. The groups were discussion-driven, rather than focused chiefly on reading from any particular philosophical canon. They were an opportunity to use philosophical techniques to investigate concepts and assumptions that are often embedded in mental health practice and broader societal discourse in a group setting.

Although those who had some previous experience of philosophy (through either formal study or personal reading) sometimes made contributions by citing the positions of philosophers (for example from the analytic, continental, and Eastern traditions), being able to do this was not essential to participation. We did not observe an obvious difference in the level of involvement from people who had no previous engagement with philosophy versus those who did. We think that this is because our approach to debate emphasised the use of philosophical techniques for assessing arguments (e.g. those described in the summary of session one), rather than learning the positions of philosophers in various canons.

We set an optional final assignment, in which participants were invited to further explore any of the content and themes of the workshop series that had particularly interested them. About half of the participants chose to present their assignment work at a final half-day meeting seven weeks after the sixth workshop. Some participants gave short presentations answering theoretical questions, drawing on the work of philosophers they had researched in their own time, and linking this work back to the core themes of the workshops. Other presentations considered practical applications of ideas from the workshops, such as how philosophy can make a positive difference to self-understanding and mental health advocacy (In fact, this paper draws insights from one such presentation given by the participant author.) One participant designed a series of conversation cards based on the philosophical questions explored in the workshops to be used to add opportunities for reflection in clinical interactions. Another was inspired to work themes from the workshop series (such as the idea of a continuum between illness and wellness) into poetry.

We, the authors, clarify that this paper does not constitute a report on a study involving human subjects, but is a reflection on the experience of the workshop series by the facilitator and a participant (and so ethical review is not applicable).

## Benefits of philosophy groups in mental health contexts

We believe there are a number of beneficial aspects of the groups in which we were involved. Our claims here represent a shared perspective we have arrived at, as a participant and facilitator (wherever we make distinctive contributions, these are signposted). We acknowledge that these experiences represent just two iterations of a discussion-driven philosophy group, and so we therefore invite researchers and mental health practitioners to consider further opportunities to investigate these potential benefits to establish whether they generalise. Readers may also find a podcast in which three of the workshop participants discuss their experiences and what they saw to be the benefits of the workshops.^5^

We think that the benefits of the groups derive chiefly from (i) the opportunity for structured discussion of experiences; (ii) the opportunity for dialogue across different mental health backgrounds; (iii) the potential to reduce stigma and to increase self-understanding and advocacy; and (iv) the potential to alleviate (some) epistemic injustice. We think these benefits are arrived at in two sorts of ways: *Content* benefits follow from engaging with the substantive philosophical content (such as contemplating different models of perceptual processing, or considering the role of irrationality in diagnostic criteria, as discussed above). *Practice* benefits follow from engaging in the practice of group philosophy, such as the dialectic exchange of ideas, and the critical methodology that enables participants to analyse claims and recognise underlying assumptions. We discuss (i)–(iv) in turn before making some further recommendations on the specifics of implementation.

### Philosophy as a means of structuring group discussion of experiences and models

Philosophy aims to provide models which enrich our understanding of the concepts that we take for granted in everyday life. This can be done through the study and critical interpretation of works in the philosophical canon, and subsequently expanding upon or responding to these works. But, as outlined in the "Philosophy groups and mental health" section, we have a different enterprise in mind: philosophy as a shared, communicative practice, in which understanding is facilitated by taking part in a dialectic that unfolds between a group of people. This sort of philosophy is, necessarily, a group activity, which is both *experience inclusive* and *model supported*, features that we will discuss in turn.

#### Experience inclusive

Introspection on, and analysis of, individual experience plays a central role in philosophical methodology, and provides a basis for constructing philosophical models. So, a central aspect of doing philosophy in a group setting is the opportunity for lived experiences of mental health to be shared and reflected upon, as well as evaluated for their underacknowledged benefits (such as providing meaning, or contributing to agency). Experience is shared on a voluntary basis, but as it is not necessary to share one’s own experience to nevertheless participate in the group dialogue, and philosophical theorising, refraining from doing so is not a detriment to participation. One can learn from the experiences of others.

#### Model supported

By this, we mean that the focus is not on isolated examples of experiences and beliefs. Instead, these are situated within a wider philosophical model according to the features that they share. This is a chance to recognise the commonalities between individuals’ experiences and beliefs, as well as an opportunity to collectively challenge existing models in accordance with which these cognitions might have previously been labelled, for instance as “mad” or “irrational” or “pathological”. For example, what does it mean for a belief to be irrational, and should we think of irrationality as indicative of mental distress or illness? Is such a label helpful or harmful? Thus, the discussion can move from individual cases to more general questions about what characteristics are necessary and sufficient for a for a label that is applied to those individual instances. Not only does this help to alleviate the pressure that can arise when concentrating on one individual’s experience as a focal point of discussion, it enables us to recharacterise our experiences according to a philosophical model which best accounts for their particular features (for instance, this could be the opportunity to situate unusual experiences within a wider model of ordinary human cognition).

From a participant perspective, we believe that the experience of engaging in a philosophy group, particularly one that meets weekly, is beneficial because it provides an ongoing connectedness which mitigates social and existential isolation. We recognise that discussion of experiences and the opportunity for connection are also fostered in peer-support and psychoeducational groups, and so we suggest that our groups share many associated *practice* benefits with these groups.

We also suggest that there are particular benefits which derive specifically from engaging with the *content* of our philosophy groups, which are not present in the abovementioned groups. Whilst some psychoeducational groups look at theoretical questions around various diagnoses and experiences of distress, they are chiefly about communicating strategies to manage or treat symptoms. And whilst the same is true of peer-support groups, they are chiefly about offering mutual support. To be clear, we think that enabling support from peers, as well as groups which impart strategies to manage distress are both important parts of mental health provision, and our proposal here is not to replace but to complement them.

Our philosophy groups have the specific aim to investigate substantive philosophical questions about mental health. The chief outcome is therefore not management or treatment, but a deepening of understanding. This is a *content* benefit that is, we argue, particular to philosophy groups. One gains a particular sort of understanding of the self after, for instance, recognising that unusual cognitions that are routinely labelled as pathological in mental health settings actually share many features with cognitions in the non-clinical population, and might even be indistinguishable on the basis of their supposed irrationality. Engaging with this content fosters a new understanding of the concepts, models, and assumptions which underlie mental health discourse and practice, bringing particular *content* benefits which we contend are not necessarily seen in groups that are already an established part of mental health provision.

One may wish to disengage from debates around rationality in mental health, finding it an unhelpful way to carve things up, but even so, sorting cognitions into the rational on the one hand, and the irrational on the other, still plays a significant role in mental health discourse and practice, for instance, by figuring in diagnostic criteria. One may disagree in principle with these labels, but can still use the tools of philosophical argument to show that even if you grant your interlocutor that these labels denote something real, still “irrational” may not demarcate something peculiar to the pathological that is not also found in people with no diagnoses or experience of ongoing mental distress.

In providing participants with philosophical tools, there are more general *practice* benefits as well. Participants can use these tools to interrogate the claim that irrational cognitions deserve to be labelled as pathological, and to assess when such cognitions are harmful and when they are helpful. In doing so, they develop general skills in critical thinking, exchange of ideas, and enquiry, which can be utilised beyond the philosophy group, (we discuss this in more detail in "Social isolation" section).

### Dialogue across different mental health backgrounds as a means of reducing stigma

The philosophy groups we facilitated and participated in were open to people with different backgrounds and experiences of mental health: people with lived experience, advocates, and mental health professionals working in different contexts. Whilst we foresee situations in which it could be beneficial to restrict participation to just one of these groups (see "Implementation considerations" section) we think there are specific benefits in running mixed groups, as we did. The groups provide a setting in which people with quite different backgrounds can come together to collectively investigate the concepts and assumptions that are often taken for granted in mental health discourse and practice, as well as hearing how their own actions based on whatever model is assumed in their practice can affect the experiences of others.

Philosophy groups proceed on the notion that the group is neutral ground, where no particular theory has primacy, and as such, all theories are open to critical questioning. We respect that there are certain power relations that arise (for instance, existing between those for whom mental health is their profession, and those without the security and authority that this bestows (Femdal and Knutsen 2017). We recommend that the group explicitly acknowledges and reflects on how these can manifest in the exchanges of those involved, and suggest that it is the responsibility of the facilitator to manage the discussion to ensure that everyone is heard (we describe how to do this in more detail in a facilitator guidance document).^6^ From a participant perspective, we do think it is possible to feel like the discussion can be had among equals, and so we emphasise the importance of the group collectively deciding the values of the space as the first activity in the first meeting, encouraging humility and self-reflection.

We report that even in mixed groups such as ours, we found a considerable amount of common ground. Philosophy can be done so as to encourage listening to the perspective of others, and imagining their experiences. Thought-experiments, a key philosophical tool, for example, often require us to imagine the experiences of others, and what these experiences might imply. So, philosophy groups constitute an opportunity for those with different backgrounds in mental health to learn from each other. Discussions that begin with a focus on a personal experience or practice can expand into an opportunity to articulate the merits and faults of different frameworks in mental health, and whether these might be changed for the better through collective action.

We can think of the benefit as coming in part from the information and ideas that are exchanged in this process, which would be a *content* benefit. There may be further benefits: existing empirical evidence on what has been called ‘intergroup contact theory’ suggests there may also be associated *practice* benefits that come from meetings of mixed groups like ours. Intergroup contact theory suggests that when people from dominant groups have meaningful engagement with those from marginalised groups, such as working together on a task towards a shared goal, the extent to which members of the dominant group stereotype those from marginalised groups (which can lead to stigma) is reduced (Alport 1955; Hewstone and Swart 2011). Various studies demonstrate that this effect holds in the case of groups delineated on the basis of mental health (Corrigan et al. 2001; Clement et al. 2012; Thornicroft et al. 2016). Participants in philosophy groups need not all agree on which theories and models are correct, but the groups do prescribe an activity which enables participants to establish a common purpose: that of investigating, challenging, and re-establishing preferable philosophical models which best capture and validate experiences of mental health. Whilst we make it clear that we did not conduct an empirical study to establish for certain whether this effect was present in our groups, we suggest that the necessary conditions for meaningful engagement are likely to have been met in our groups, for intergroup contact benefits to ensue. We gladly invite further empirical research on the topic.

### Decreased self-stigma, increased self-understanding and advocacy

Given the pervasive stigma to which people with experiences of mental distress and mental illness are exposed, we agree with other authors (cited in "Background" section) that the onus is on those in positions of power to take measures to avoid stigmatising language and behaviour in their interactions with those experiencing mental distress. That said, “self-stigma”, in which people internalise and view aspects of their social identity through the lens of stereotypes embedded in dominant cultural narratives, is a real and deleterious phenomenon, affecting people experiencing mental distress (Corrigan et al. 2014). These attitudes may affect whether people seek help: one study shows that as attitudes of mental health self-stigma increase, attitudes towards treatment become more negative (Sickel et al. 2019). This effect has also been seen in mental health professionals who are experiencing distress (in Knaak et al. 2017). Help-seeking is affected by the extent to which mental health stigma is a cultural norm: in countries in which mental health stigma is more prevalent in the population, individuals are less likely to seek support for their mental health (Bracke et al. 2019), and so efforts to reduce stigma in general of course remain important (as we emphasise in the previous subsection).

From a participant perspective, we believe that philosophical exploration facilitates self-understanding. As we just saw, group philosophy is an opportunity to challenge external descriptions of our experiences, and to recharacterise them in a way that better captures their different features, including how they can hinder us, but also how they can help us (benefits deriving from engaging in the substantive *content* of the philosophy group). Relatedly, from a participant perspective, taking part in philosophical discussion can enable us to build confidence in expressing ourselves, helping us to defend our viewpoints and better advocate for our position (a *practice* benefit). This applies to how we describe and communicate about our own situation. Philosophy also provides tools for understanding the dynamics of the mental health institutions and broader societal structures that we encounter, and for advocating for change within these structures. Participants also involved in advocacy can draw on learning from philosophy groups to inform their own actions aimed at institutional change (such as contributions to committees of medical establishments, and reviews of mental health legislation, etc.).

Alleviating stigma is complex, and evidence suggests that interventions which aim to normalise mental distress and the associated unusual cognitions can actually increase attitudes that sufferers are thereby somehow responsible for their condition (e.g. Gergel 2014). We think it is therefore important to include case studies (as we did) which give participants an opportunity to reflect on how mental distress can often be contextualised in the life experiences, political and economic circumstances, and social relationships of the individuals involved, to challenge the narrative that people are responsible for their own distress.

### Potential to alleviate epistemic injustice

Following our arguments in the subsections “Philosophy as a means of structuring group discussion” and “Dialogue across different mental health backgrounds”, we suggest that doing philosophy in mixed groups could have the effect of (somewhat) alleviating testimonial epistemic injustice, because mental health professionals will be able to learn from people with lived experience who may offer examples of being on the receiving end of testimonial injustice, such as the experience of Richard Lakeman (2010) summarised in "Background" section. We must be wary of expecting people with lived experience of testimonial injustice to do the bulk of the epistemic labour by obligating them to share their experiences. However, if the groups are run as we suggest in the facilitator guidance we produced, and the group is a safe and non-judgemental space, stories may well be shared organically. Even if the group does not have access to personal stories like these, mental health professionals still have the opportunity to critically investigate the assumptions underlying mental health practice through engagement with the substantive philosophical content (as described in “Philosophy as a means of structuring group discussion”). Reflecting on this content, such as whether notions of irrationality should be decoupled from notions of pathology, may go some way towards helping participants refrain from devaluing testimony from people with lived experience of mental distress. As Crichton et al. (2017) recommend, learning about the phenomenon of epistemic injustice, and the background conditions which produce it, is essential to helping to alleviate it in clinical settings.

Following on from the previous subsection, we suggest that philosophy groups have the potential to (somewhat) contribute to restoring hermeneutic epistemic justice for people with lived experience. This is because they provide the opportunity to develop a philosophical account of experiences of mental distress which incorporates their deeply meaningful and positive features, rather than focusing only on negative attributes (thus drawing on the *content* of the groups). As we saw in Sect. 1, these sorts of hermeneutic resources for self-understanding are not always readily available from drawing on popular culture and public discourse about mental health. From a lived experience perspective, we emphasise that living with mental distress can involve very deep and fundamental questioning of one’s sense of self, and this can lead to feelings of alienation. We think that the *practice* of philosophy is effective at reaching these profound levels of experience, as well as providing a means to articulate them.

We recognise that the factors which account for epistemic injustice in mental health are deeply entrenched, and many strategies are required to restore justice (Crichton et al. 2017; Sanati and Kyratsous 2015; Wardrope 2015; Kurs and Grinshpoon 2018; Miller Tate 2018). As such, we propose that philosophy groups constitute a modest, but potentially significant, strategy for change to use in conjunction with existing strategies.

## Implementation considerations

We recognise that multiple factors may affect participants’ experiences of the groups, such as the content discussed, the manner in which they are facilitated, and the setting. We therefore encourage interested mental health practitioners and peer-support group facilitators to consider the possibility of philosophy groups in mental health contexts with which they are familiar, and to bring their own expertise to bear on setting them up. Exploratory initiatives such as running an experimental one-off group, or giving potential participants the opportunity to make suggestions about what they would want from a philosophy group in a questionnaire could be informative.

Philosophy groups of the kind we envisage have an agile, flexible format. They can be resource-light, and adapted to run in a number of different settings. As mentioned above, in developing our own workshops, we have developed open-access resources for facilitating such a group, which do not assume any previous philosophical experience, which can be adapted in accordance with the facilities and equipment available, use of a room and some chairs is sufficient. These resources can be accessed online*.*^7^ Whilst prior philosophical education may not be necessary, for the reasons discussed in “Dialogue across different mental health backgrounds”, we would recommend that the person leading the sessions has some training specifically regarding facilitating marginalised groups, in order to create a space in which everyone is comfortable and able to participate (e.g. valuing anonymity, mutuality and peer-support). This sort of training is often available through mental health charities.

The relatively agile format of philosophy groups, and the fact that they are not tied to the values and practices of any particular institution, means that they can be run in a wide variety of settings. This could be, for instance, in partnership with a mental health advocacy group as we did; in in-patient wards or alongside other NHS services (such as CBT groups); in local community centres; or even as “pop-up” sessions in a coffee shop or other public space (although it should be considered how anonymity would be managed in a public space).

We have discussed our experience of groups that involve people with different relationships to mental health, but we foresee situations in which one might want to run groups with (for example) people with lived experience only, or with clinicians only. This might alter the focus of the group. A group of mostly clinicians might want to focus on addressing philosophical claims embedded in clinical practice, such as how the notion of irrationality is used in diagnostic criteria, and safeguarding against testimonial injustice in clinical practice. A group involving only people with lived experience might wish to focus on developing shared resources for advocacy and understanding.

Bonny Astor, one of the participants from our group, has since run a series of philosophy sessions, based on the content and methodology used in our original groups, in Pentonville Prison (UK). There are distinctive challenges in the prison setting, including lack of participant continuity (the nature of prison life is such that inmates are not always at liberty to attend sessions consistently), meaning that it was harder to have a series of sessions which build on material discussed in the previous weeks. In this case, the facilitator adapted the material to the new context, keeping the core themes and ideas, and leaving out some of the more theory-laden aspects. However, she reports that thought experiments and the use of concrete examples worked well as a means of opening up the discussion and leading on to the more theoretical models in each individual session.

## Conclusion

We saw there to be a range of benefits to running discussion-driven philosophy groups for people with a range of backgrounds in mental health, and we have provided some interpretations of these benefits. We maintain that: (i) there is value in engaging with substantive philosophical investigation of the concepts and assumptions underlying mental health discourse, particularly when this is a structured, communicative practice; (ii) philosophy groups present the opportunity for dialogue across different mental health backgrounds and this may go some way towards alleviating mental health stigma; (iii) that such groups can reduce self-stigma and increase self-understanding and advocacy; and (iv) they offer the potential to (somewhat) alleviate epistemic injustice. We urge interested mental health practitioners and peer-support group facilitators to consider whether a philosophy group in their local context could bring similar benefits.

## Footnotes

1 There are different frameworks for understanding the experiences and cognitions that often lead to diagnoses of mental illness. For instance, there are frameworks which defer to medical explanations, according to which these experiences are the result of biological processes, and are amenable to medical interventions (Mechanic 1999; Wardrope 2015). Then there are frameworks which defer to social explanations, and which locate understanding of these experiences in past trauma, relationships, and material circumstances (Mulvany 2001; Beresford et al. 2010). Then there are hybrid frameworks which emphasise a combination of factors (Pilgrim 2002; Johnstone and Boyle 2018). In this article we do not presuppose a particular framework, and refer instead to “the experience of mental distress”, or “cognitions which attract a psychiatric diagnosis”. Sometimes when we describe a particular study, we adopt the language of that study’s authors for the purpose of communicating their findings, but in doing so we do not thereby adopt their preferred framework. This article describes a workshop series in which we encouraged participants to explore the available frameworks, and adopt (or to develop) the framework that they felt best captured their experiences, and we welcome the reader to do this as well.

2 The original proposal was for 60 min sessions, with the following half-hour reserved in case participants wanted to continue discussion, but early on, both groups discussed and decided that they would like the sessions to run for the full 90 min.

3 Open access resources for the Philosophy of Mind Workshop Series can be found here: https://www.birmingham.ac.uk/generic/perfect/resources/philosophy-of-mind.aspx

4 Examples were drawn from cases studied in the course of the first author’s research project, on the Pragmatic and Epistemic Role of Factually Erroneous Cognitions and Thoughts (Project PERFECT). Further information on PERFECT is available here: http://www.projectperfect.eu/.

5 Further information on the podcast, and a link to listen, can be found here: http://www.canstream.co.uk/camden/index.php?id=970

6 The facilitator guidance document can be found here: https://www.birmingham.ac.uk/Documents/college-artslaw/ptr/perfect/philosophy-of-mind/Facilitator-Notes.pdf

7 See footnote 2 for a link to the open access resources.
